# Notes and News

**Published:** 1892-04-09

**Authors:** 


### Notes and News.

COLLECTED AND COMMUNICATED.

We should like to know what scientists would suggest in the case of the boy delinquent who tampered with his medical certificate. The doctor gave him leave of absence from school for "3 or 4" days, which he converted into 304 days! The case is all the more difficult because, although the magistrate treated the matter with becoming seriousness, the "Court" are reported to have taken the whole thing as a joke. Such qualities as were displayed in the boy's action are not incapable of being utilised in a right direction, but it seems to us that justice defeats its own ends by prosecution for such offences in open court.

In our crowded cities, where the majority of the poorer population hardly enjoy a breath of pure air from one year's end to another, there must necessarily be a large amount of disease of the respiratory organs. The amount of medical relief required is enormous, far too great, indeed, to cope with, except to a very limited extent. The City of London Hospital for Diseases of the Chest, of which Queen Victoria is Patron, is doing a work of untold benefit. Since the year 1855, no less than 25,500 in-patients have been received. Last year the number admitted was 1,236. The out-patients numbered 15,529. Like most of the Metropolitan hospitals, this institution is sadly in want of funds. The annual subscriptions only amount to £4,500, to meet an expenditure of £10,000. This leaves a very large deficiency to be made up by donations and bequests, a precarious form of income. There remains, besides the necessary sum for current expenses, a deficit of £3,000 on last year's accounts to be provided for. The hospital holds its annual dinner on May 24th, when it is hoped that many new and old friends will make special efforts to place this excellent charity on a secure footing. The sanitary arrangements and ventilation of the building are undergoing alteration and reconstruction, which are much needed, and for this another £3,000 will be required. The treatment of chest complaints is expensive, whilst the benefit of hospital care to the patients is very apparent.

Mr. E. Brodie Hoare, M.P., who presided at the Festival Dinner of the Hampstead Home Hospital at the Hotel Metropole on March 29th, admirably summed up the work of this institution when he said that the poor when they fell sick were provided for by the great hospitals of the metropolis, but there was a very large class to whom nursing an illness at home was almost an impossibility. All knew the small home where quiet was rendered impossible by the baby in the nursery or the people next door. The cries and noise which could not be avoided were a very serious strain upon the sick, and often acted as a retarding element in their recovery. Home hospitals were one of the best developments of the times, as there, for a moderate consideration, such sick persons could be nursed. The very devoted and able Honorary Secretary (Mr. R. A. Owthwaite) announced during the evening that over £1,000 had been received as the result of the dinner.

The difficulties of combined rate and voluntary-support for hospitals is illustrated in the case of the Cork Fever Hospital. The institution in question is only more fortunate than some others in having at least a portion of the sum of money it urgently needs, granted without that tedious delay which frequently occurs. The application recently made to the Cork Corporation was for £1,000 towards maintenance and support. The High Sheriff, on behalf of the Trustees of the hospital, represented that they had been going into debt for the past three years, because the Corporation had curtailed their former subscription. The debt amounted to £579, and £400 was absolutely necessary for repairs. After a lengthy discussion, one Alderman stated that in case of emergency or epidemic a grant would be forthcoming. The High Sheriff remarked that it might come too late; to which the Alderman replied that money could do anything. After this enlightened remark, £300 was unanimously voted at the suggestion of the Mayor! If the voluntary hospitals have a difficult task to make both ends meet, matters are frequently far worse with these rate-supported institutions. They are not likely to become the "pets of the public" at any time, and they suffer in the same way as kindred institutions in far-off India, where an increasing endeavour to place hospitals under local government has in most cases been attended with, at any rate, a temporary falling off in private subscriptions and interest.

Those who were present at the festival dinner of the Hospital for Sick Children, recently given at the Hotel Metropole, must have been much struck by the admirable speech made by the Lord High Chancellor. He remarked that "No class of institution appealed so strongly to their sympathies as that class which had to tend the suffering of sick childhood." Further he said, "One pathetic pitiful statement had been made to him, and had affected him very much, and that was, that for every one little child admitted into the hospital, three must be rejected, because there was not room to deal with them. What an amount of terrible suffering, and so far as his personal experience went, of patient and uncomplaining suffering, this represented. The hospital asked for £10,000 to complete its enlargement, and he thought there should be no difficulty whatever in raising that money. He urged all present to interest their friends in this excellent charity, so that the discredit should not rest upon the metropolis that such an institution as the Hospital for Sick Children urgently requiring funds." The Lord Chancellor in his speech, also commented on the amazing fact that a very large portion of the dwellers in the metropolis did not assist to maintain the hospitals at all. Mr. Adrian Hope announced the splendid sum of £4,595 in the form of donations, annual subscriptions, building fund subscription and endowment, as a result of the festival.

---

The case of Patrick Dunn, who died at St. Bartholomew's Hospital on the 27th ult., of cerebal apoplexy, after being twice refused admission by one of the house surgeons, who thought the man to be dead drunk, graphically illustrates a grave defect in the administration of our metropolitan hospitals. These cases, which are familiarly known as drunk or dying, recur at irregular intervals, and invariably shock, and rightly shock, the public conscience. They occur because it is the practice at the large London hospitals to leave the admission of emergency cases in the hands of senior students, who, in most cases, have only recently obtained a medical qualification. To deal with them properly a wide experience in diagnosis is essential. The governing committees and medical boards ough. at once to draw up a few stringent rules which should provide that where cases of supposed drunkenness, like that of poor Patrick Dunn, are brought to the hospital they should not be permitted to leave it until the following morning. It is, of course, undesirable to admit cases of the kind into the ordinary wards, but in the provinces it is customary to set apart a certain number of casual beds for the accommodation of these cases, and so all possibility of mistaken diagnosis and grievous scandal is removed. Where a patient is really drunk, his admission to an ordinary casualty ward would be unfair to the other inmates, who might be seriously disturbed by his presence, or subjected to disagreeables, for obvious reasons. The casual beds we allude to are, therefore, placed in the basement, or some isolated portion of the buildings, where no harm can result, and the patient can be properly watched The continuance of the present system by the managers of the large London hospitals is indefensible. It is very unfair to the young house surgeons (who, by the way, are not paid, as one of our lay contemporaries erroneously states), to the best interests of the hospitals, to the public, and to the patients, and demands an immediate alteration. We believe that cases of delirium tremens are admitted to the casualty wards at St. Bartholomew's, and if this be so, it is a striking illustration of the necessity of adopting definite regulations to secure the retention of doubtful cases. Our experience proves that no such case as Patrick Dunn's need arise at any well-regulated hospital, for which reason, although we yield to no one in our admiration of the excellent work done by the voluntary hospitals, we are bound to declare that the authorities of any hospital where a similar case occurs in future ought to be held responsible for the consequences.

